# Professor Charles Frick

**Published:** 1860-04

**Authors:** 


### NECROLOGY.

*Professor Charles Frick.*—It is with a grief we are wholly unable to express that we announce to our readers the untimely death of the distinguished Professor of Materia Medica and Therapeutics at the University of Maryland, Dr. Charles Frick. So suddenly has this calamity fallen upon us, that we are still scarcely able to regard it as anything but a hideous dream, from which we long to awake.

He fell in the faithful discharge of his duty, and has thus added another name to the muster-roll of that "noble army of martyrs" who have sacrificed themselves to save the lives of those entrusted to their care. The circumstances of the case were these: A poor negro woman had contracted *diph-*

*therite*, or malignant sore throat, and was sent to the Infirmary attached to the University of Maryland. Dr. Frick, being in charge, gave her the only chance for life by performing the operation of tracheotomy. Most unfortunately, he contracted the disease himself. The operation was performed on Tuesday, the 20th of March. On Wednesday the Doctor himself was taken ill, and by Sunday morning, such was the extension of the false membrane into the larynx and trachea, that he was only saved from immediate asphyxia by the same operation. The larynx was opened early in the morning, and the patient experienced some relief. Unhappily, however, this was but of short duration, as he sank and died about noon of the same day, the 25th of March.

Thus perished, in the prime of his life, in the full career of usefulness, one of the most eminent of our medical men. The chasm he has left it will be difficult if not impossible to fill. A skillful and observant physician, who took broad, philosophical views of his profession, and thought profoundly and originally upon the various questions of medical science and art, his loss as a teacher will be most acutely felt by those young men he labored so zealously and successfully to instruct. The death of such a man exerts its influence far beyond the immediate time of his decease. Those habits of thought his example and precept were so sure to inculcate upon the students who listened to his expositions of disease and remedies, can only be acquired by careful training under an original thinker like himself. Not only the medical profession, however, but the entire community feels his loss. His death has cast a profound gloom over the whole city. His native amiability and goodness of heart won the love of all who were so happy as to know him intimately, while his graceful and attractive manners secured the admiration of the most casual acquaintance. It is doubtful if he ever had an enemy—certainly he never deserved one. Of him we can most emphatically say,

> **"None knew him but to love him,**
> **None named him but to praise."**

---

It was our intention to give a fuller account of the life and labors of this amiable and accomplished gentleman, but not having at present a complete list of his contributions to medical science, we shall defer it until July.

